# Pulsed high-dose dexamethasone modulates Th1-/Th2-chemokine imbalance in immune thrombocytopenia

**DOI:** 10.1186/s12967-016-1064-9

**Published:** 2016-10-24

**Authors:** Zongtang Liu, Meiying Wang, Shufen Zhou, Ji Ma, Yan Shi, Jun Peng, Ming Hou, Chengshan Guo

**Affiliations:** 1 Medical College, Shandong University, Jinan, 250012 China; 2 Department of Hematology and Oncology, Tancheng Hospital, Linyi, 276100 China; 3 Department of Rheumatology and Immunology, Peking University Shenzhen Hospital, Shenzhen, 518000 China; 4 Department of Rheumatology and Immunology, the Affiliated Shenzhen Baoan Hospital of Southern Medical University, the 2nd Longjing Road, Baoan, Shenzhen, 518101 China; 5 Department of Hematology, Qilu Hospital, Shandong University, Jinan, 250012 China; 6 Shenzhen Research Institute of Shandong University, Shenzhen, 518057 China; 7 Department of Rheumatology, the Affiliated Shenzhen Baoan Hospital of Guangdong Medical University, Shenzhen, 518101 China

**Keywords:** Immune thrombocytopenia, Chemokine, Chemokine receptor, Th1 polarization, High-dose dexamethasone

## Abstract

**Background:**

Chemokines and chemokine receptors play important roles in autoimmune diseases; however, their role in immune thrombocytopenia (ITP) is unclear. High-dose dexamethasone (HD-DXM) may become a first-line therapy for adult patients with ITP, but the effect of HD-DXM on chemokines in ITP patients is unknown. Our aim was to investigate the mechanism of pulsed HD-DXM for management of ITP, specifically regarding the chemokine pathways.

**Methods:**

Th1-/Th2-associated chemokine and chemokine receptor profiles in ITP patients before and after pulsed HD-DXM was studied. Plasma levels of CCL5 and CXCL11 (Th1-associated) and of CCL11 (Th2-associated) were determined by ELISA. Gene expression of these three chemokines and their corresponding receptors CCR5, CXCR3, and CCR3, in peripheral blood mononuclear cells (PBMCs) was determined by quantitative RT-PCR.

**Results:**

Thirty-three of the thirty-eight ITP patients responded effectively to HD-DXM (oral, 40 mg/day, 4 days). In ITP patients, plasma CXCL11 levels increased, while CCL11 and CCL5 decreased compared to controls (*P* < 0.05). Similarly, gene expression of CXCL11 and its receptor CXCR3 increased, while CCL11 and CCR3 decreased (*P* < 0.05). CCL5 expression did not significantly change; however, expression of its receptor CCR5 increased (*P* < 0.05). Interestingly, in the patients who responded to pulsed HD-DXM, CXCL11 and CXCR3 expression was down-regulated, while CCL11 and CCR3 expression was up-regulated (*P* < 0.05). Meanwhile, CCL5 expression was up-regulated and CCR5 was down-regulated by HD-DXM (*P* < 0.05).

**Conclusions:**

The abnormal profiles of Th1-/Th2-associated chemokines and chemokine receptors may play important roles in the pathogenesis of ITP. Importantly, regulating Th1 polarization by pulsed HD-DXM may represent a novel approach for immunoregulation in ITP.

## Background

Immune thrombocytopenia (ITP) is an acquired autoimmune-mediated bleeding disorder, mainly characterized by anti-platelet antibody-mediated thrombocytopenia in the reticuloendothelial system [1]. Interestingly, it has been shown that the destruction of platelets is associated with enhanced T-helper 1 (Th1) expression and an elevated Th1/Th2 ratio in ITP patients [2, 3]. However, expression of chemokines and chemokine receptors associated with the Th1/Th2 imbalance, particularly relating to immune regulation by modified application of glucocorticoids, has yet to be explored.

Chemokines and their receptors are involved in cellular migration and proliferation, molecule adhesion, regulation of apoptosis, T cell differentiation, leukocyte trafficking, and cytokine production [4]. For example, chemokine CCL5 is not only required for attracting CCR5^+^ T cells but is also essential for their activation [5]. CCR5 is specifically expressed on Th1 cells and has been reported as a critical chemokine receptor in several autoimmune diseases with polarization of Th1 cells such as rheumatoid arthritis, multiple sclerosis, Crohn’s disease and oral lichen planus [6–8]. CXCL10 expression is essential for Th1 cell differentiation [9, 10]. CXCL11 is overexpressed in Th1-dominated skin disorders, such as, psoriasis, lichen planus, and atopic dermatitis [11–13]. Moreover, CXCL11 is the most potent chemoattractant and has the highest receptor binding affinity for CXCR3 [14]. CXCR3, a marker for activated T lymphocytes, is characteristically expressed on Th1 and CD8^+^ T cells [15, 16] and is required for generation of interferon-γ (IFN-γ) -secreting Th1 cells in vivo [9]. In contrast, CCR3 is predominantly expressed on Th2 cells [17]. Recently, by characterizing Th1-associated chemokine receptors CXCR3 and CCR5, and Th2-associated chemokine receptor CCR3, we demonstrated that the abnormal expression of Th1/Th2 chemokine receptors may participate in the splenic immune dysregulation in ITP patients [18].

Glucocorticoid therapy is still the primary choice for treatment of ITP. Due to the poor long-term sustainability and possible adverse effects of long-term administration of corticosteroids [19], recent reports suggested that high-dose dexamethasone (HD-DXM) might be a promising regimen to replace the classical prednisone therapy [20–22]. Here, we investigated Th1- and Th2-associated chemokines and their receptors in peripheral blood of ITP patients. Specifically, we investigated whether HD-DXM could rectify the abnormal Th1-/Th2-associated chemokine and chemokine receptor profile in ITP.

## Methods

### Patients and controls

Between December 2014 and August 2016, 38 patients (12 males and 26 females, age range 12–71 years, median age 40.5 years) with active ITP in Shenzhen Baoan Hospital Affiliated to the Southern Medical University and the Second Hospital of Shandong University were enrolled. Patients’ platelet counts ranged from 2 to 34 × 10^9^/L, with a median count of 10 × 10^9^/L. All patients required treatment because of clinically significant bleeding. All of the cases met the diagnosis criteria of ITP as previously described [23]. None of them had received any corticosteroid or immunosuppressive therapy within the 3 months prior to sampling. Patients with diabetes, hypertension, cardiovascular diseases, pregnancy, active infection, or connective tissue diseases, such as systemic lupus erythematosus, were excluded. The clinical features of the patients are shown in Table 1.

**Table 1:** Clinical characteristics of patients with ITP

Patient	Gender	Age	Platelets (×10^9^/L)	Bleeding symptoms	Major therapy	Platelets (×10^9^/L)
			Before treatment			After treatment
1	F	54	23	EC	GC	118
2	F	37	6	EC, PT	GC	154
3	F	23	15	EC, EP	GC	98
4	F	58	18	EP, GUH	GC	222
5	F	48	3	EC, GUH	GC	245
6	M	67	12	PT	GC	97
7	F	40	10	PT	GC	56
8	M	59	4	EC	GC	234
9	F	60	27	EC, GUH	GC	67
10	M	70	10	EC, PT	GC	21
11	M	49	9	PT	GC	180
12	M	12	34	EC	GC	78
13	F	13	21	EC	GC	203
14	F	26	10	EC, GIH	GC	82
15	F	28	19	EC	GC	168
16	M	65	20	EC, GIH	GC	166
17	M	42	11	PT	GC	267
18	F	17	9	EC	GC	80
19	F	56	2	PT	GC	9
20	M	61	27	PT	GC	174
21	F	63	10	EC, GH	GC	199
22	F	50	14	EC, PT	GC	143
23	M	31	11	EC	GC	20
24	F	26	25	PT	GC	156
25	F	71	21	PT	GC	151
26	F	51	28	EC, GUH	GC	32
27	F	28	8	PE, PT, ET	GC	103
28	F	24	9	EC, EP	GC	197
29	F	29	16	PT	GC	173
30	F	34	22	PT	GC	129
31	F	41	7	PE, PT	GC	301
32	F	20	5	EC, GUH	GC	24
33	M	25	23	PT	GC	71
34	M	37	3	PT	GC	96
35	F	19	2	EC, PT	GC	136
36	M	33	7	PT	GC	84
37	F	46	16	PT	GC	183
38	F	42	10	PT	GC	158
Median		40.5	10			148.5
Range		12–71	2–34			9–301

*EC* ecchymoses; *GC* glucocorticoid; *PT* petechiae; *EP* epistaxis; *GUH* genitourinary hemorrhage; *GIH* gastrointestinal hemorrhage; *GH* gingival hemorrhage

The control group consisted of 31 healthy adult volunteers (11 males and 20 females, age range 21–53 years, median 31.5 years), whose platelet counts ranged from 148 to 342 × 10^9^/L, with the median count of 196 × 10^9^/L.

### Treatment regimen

Dexamethasone (DXM, 40 mg/day) was administered orally for four consecutive days. No maintenance or other treatment modality was used. Initial response was evaluated 2 weeks after treatment initiation. An effective response was defined as a platelet count greater than 30 × 10^9^/L, at least a twofold increase compared to baseline, and an absence of bleeding [24].

### Reagents and antibodies

Lymphoprep was obtained from Hao Yang Biological Manufacturer, Tianjin, China. Human CCL5, CXCL11, and CCL11 ELISA kits were purchased from R&D Systems, USA. TriZol reagent for total RNA was provided by Invitrogen, CA, USA. PrimeScript™ RT reagent Kit, SYBR Premix Ex Taq TM II were supplied by TaKaRa, Japan. Oligonucleotide primers for real-time PCR for CCL5, CXCL11, CCL11, CCR5, CXCR3, CCR3, and β-actin were synthesized by TaKaRa, Japan. Phosphate-buffered saline, fetal bovine serum, trypsin–EDTA and RPMI 1640 was provided by Gibco, USA. The microplate reader was supplied by Thermo Scientific, USA.

### PBMCs and plasma preparation

Peripheral blood mononuclear cells (PBMCs) were purified from heparinized venous blood samples immediately following collection using Lymphoprep density gradient centrifugation. Plasma was isolated from fresh samples by centrifugation and stored at −80 °C until use.

### Chemokine secretion

Plasma concentrations of CCL5, CXCL11, and CCL11 in active patients, patients who responded to HD-DXM, and controls were evaluated using a matched ELISA kit. All procedures were performed according to the manufacturer’s instructions. Each sample was tested in duplicate and optical densities were read using a microplate reader.

### Assay of chemokine mRNA expression

Total RNA was extracted from PBMCs with TriZol according to the manufacturer’s instructions. cDNA was reversely transcribed from total RNA using an eppendorf Realplex2 PCR Detection System and the PrimeScript™ RT reagent kit. Following the manufacturer’s protocol, real-time PCR was performed to determine the expression of chemokine mRNA with SYBR Premix Ex Taq™ II. Reverse transcription was carried at 42 °C for 5 min. PCR was performed as follows: initial denaturation 30 s at 95 °C, and for 40 cycles of 5 s at 95 °C for denaturation and 30 s at 60 °C for transcription. All samples were measured in triplicate. The amount of chemokine mRNA was normalized with β-actin and expressed as relative quantification (RQ): RQ = 2^−ΔΔct^. The primer sequences for CCR5, CXCR3, CCR3, CCL5, CXCL11, CCL11, and β-actin are listed in Table 2.

**Table 2:** Primer sequences

Target gene	Forward (5′-3′)	Reverse (5′-3′)
CCL5	CAGAGAAGAAATGGGTTCGGGA	GAGCGGGTGGGGTAGGATAGTG
CXCL11	GCCTTGGCTGTGATATTGTGTGC	CATCGTTGTCCTTTATTTTCTTT
CCL11	TCAGCGACTAGAGAGCTACAGGA	GCTTTGGAGTTGGAGATTTTTGG
CCR3	TACACAGGAATCATCAAAACGC	AGGAAGAGAGAAGGATAGCCAC
CXCR3	CAACGCCACCCACTGCCAATAC	CAAAGGCCACCACGACCACCAC
CCR5	GAAGAGCTGAGACATCCGTTCC	ACACCAG TGAGTAGAGCGGAG
β-actin	CACTCTTCCAGCCTTCCTTCC	AGGTCTTTGCGGATGTCCAC

### Statistical analysis

The Statistical Package for the Social Sciences version 19.0 (SPSS Inc., Chicago, IL) was used for statistical analysis of experimental data. Values are presented as mean ± SD. The date of plasma chemokine levels and mRNA levels among the active ITP groups and normal controls were normally distributed. The comparison between two groups was conducted by *t* test, measurement data were compared by ANOVA with Bonferroni test analysis among multiple groups. Briefly, ANOVA analysis was for comparison among active ITP, normal controls, and responders, and Bonferroni test was for comparison between any of two groups. Values of *P* < 0.05 were considered statistically significant.

## Results

### Therapeutic effect of HD-DXM

Out of all 38 patients, 33 cases (10 males and 23 females, median age 38.5 years, range 12–71 years) responded effectively to the HD-DXM therapy, according to the standard definition [19]. In these responders, platelet counts ranged from 56 to 301 × 10^9^/L, with a median count of 155 × 10^9^/L, as shown in Table 1. No bleeding or other complications were apparent, such as metabolized abnormality of multiple systems. Withdrawal symptoms were not observed throughout the treatment either.

### Plasma levels of CCL5, CXCL11, and CCL11

**Fig. 1:**
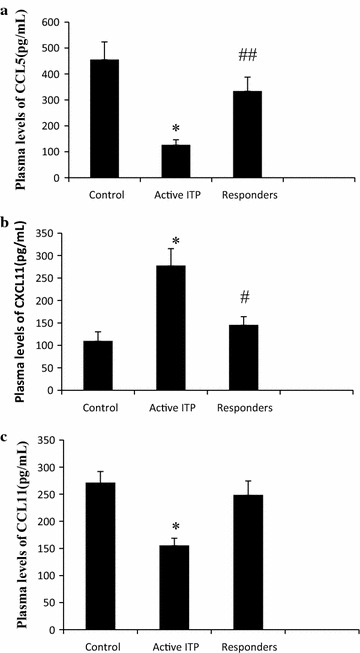
Plasma CCL5, CXCL11, and CCL11 levels. **a** Compared to normal controls, CCL5 levels were reduced in the active ITP. In responders (ITP patients who responded well to HD-DXM), CCL5 improved significantly but still lower than control (*P* = 0.000 < 0.05). **b** Compared to control, CXCL11 levels were increased in active ITP patients (*P* = 0.000 < 0.05). In responders, CXCL11 levels decreased, but were still higher than controls (*P* = 0.018). **c** Compared to control, CCL11 levels in active ITP patients were lower than in controls (*P* = 0.000 < 0.05). Responders had significantly higher CCL11 levels than active ITP patients that were not significantly different from control (*P* = 0.255 > 0.05). *Asterisk* represents *P* < 0.01 vs control and responders, ^#^ *P* < 0.05 and ^##^ *P* < 0.01 vs control

Compared to normal controls, plasma CCL5 levels were reduced in the active ITP group (126.18 ± 19.84 vs 455.54 ± 68.29 pg/mL). Plasma CCL5 in ITP patients who responded well to HD-DXM (responders) improved (334.08 ± 53.63 pg/mL) but was still lower than control (Fig. 1a).

Compared to control, CXCL11 levels were increased in active ITP patients (277.84 ± 37.78 vs 109.83 ± 20.30 pg/mL). In responders, CXCL11 levels decreased (145.53 ± 18.44 pg/mL), but were still higher than controls (Fig. 1b).

Compared to control, CCL11 levels in active ITP patients were lower than in controls (155.60 ± 13.22 vs 271.47 ± 20.48 pg/mL). Responders had significantly higher CCL11 levels (248.88 ± 25.53 pg/mL) than active ITP patients that were not significantly different from control (Fig. 1c).

### CCL5, CXCL11, and CCL11 mRNA expression

**Fig. 2:**
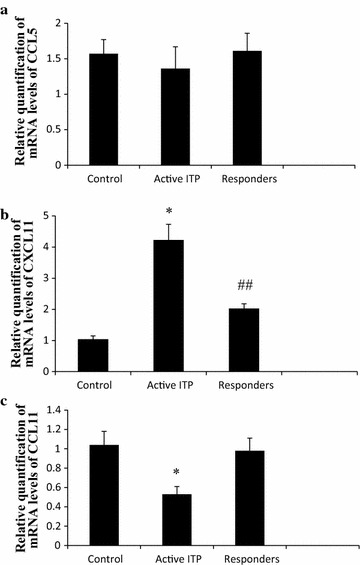
Relative quantification of mRNA levels of CCL5, CXCL11 and CCL11. **a** No significant difference was found between any groups (*P* = 0.382, 0.390, 1.000 > 0.05, respectively). **b** Compared to normal controls, CXCL11 mRNA levels were increased in active ITP patients (*P* = 0.000 < 0.05). CXCL11 expression in responders was significantly reduced compared to active ITP, but still higher than controls (*P* = 0.000 < 0.05, respectively). **c** Compared to control, CCL11 mRNA levels in active ITP patients was reduced (*P* = 0.000 < 0.05), however, CCL11 mRNA levels improved in responders and was not significantly different from control (*P* = 1.000 > 0.05). * *P* < 0.01 vs control and responders, ^##^ *P* < 0.01 vs control

Chemokine mRNA levels in PBMCs were determined by qRT-PCR. For CCL5 expression, no significant difference was found between any group (1.36 ± 0.31 vs 1.57 ± 0.20 vs 1.61 ± 0.25, in active ITP, control, and responders. Fig. 2a).

Compared to normal controls, CXCL11 mRNA levels were increased in active ITP patients (4.23 ± 0.50 vs 1.04 ± 0.11). CXCL11 expression in responders was significantly decreased (2.03 ± 0.15) compared to active ITP, but still higher than controls (Fig. 2b).

Compared to control, CCL11 mRNA levels in active ITP patients was reduced (0.53 ± 0.08 vs 1.04 ± 0.14). However, CCL11 mRNA levels improved in responders and was not significantly different from control (0.98 ± 0.13) (Fig. 2c).

### CCR5, CXCR3, and CCR3 mRNA expression

**Fig. 3:**
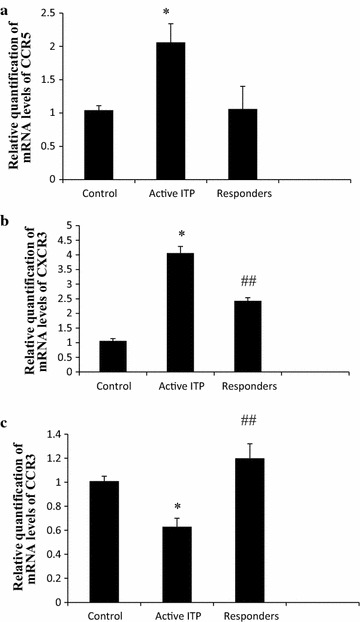
Relative quantification of mRNA levels of CCR5, CXCR3, and CCR3. **a** Compared to control, CCR5 mRNA levels in active ITP patients were elevated (*P* = 0.000 < 0.05). In responders, CCR5 expression returned to normal levels (*P* = 1.000 > 0.05). **b** Compared to control, CXCR3 mRNA levels in active ITP patients were elevated (*P* = 0.000 < 0.05). CXCR3 expression in responders decreased (*P* = 0.000 < 0.05), but was still higher than that in control (*P* = 0.000 < 0.05). **c** Compared to control, CCR3 mRNA levels in active ITP decreased (*P* = 0.000 < 0.05); after treatment with HD-DXM, CXCR3 expression in responders increased (*P* = 0.000 < 0.05). * *P* < 0.01 vs control and responders, ^##^ *P* < 0.01 vs control

Compared to control, as mRNA expression of the Th1-associated chemokine receptors, both of CCR5 and CXCR3 were elevated in active ITP patients (2.05 ± 0.20 vs 1.03 ± 0.06, 4.06 ± 0.23 vs 1.06 ± 0.08, respectively) (Fig. 3a, b). In responders, CCR5 expression returned to normal levels (1.06 ± 0.15, Fig. 3a). CXCR3 expression in responders decreased (2.43 ± 0.11), but was still higher than that in control (Fig. 3b).

Compared to control, as one of the Th1-associated chemokine receptors, CCR3 mRNA levels in active ITP decreased (0.63 ± 0.07 vs 1.01 ± 0.04); after treatment with HD-DXM, CXCR3 expression in responders increased significantly (1.20 ± 0.12, Fig. 3c).

## Discussion

ITP is an immune-mediated acquired disease characterized by a persistent or transient decrease of platelet count. In this study, we found an abnormal profile of Th1- and Th2-associated chemokines and their receptors in peripheral blood of ITP patients. Further, we demonstrated that HD-DXM can rectify the abnormal Th1-/Th2-associated chemokine and chemokine receptor profile in ITP.

Our findings showed that 85.71 % of ITP patients responded well to pulsed HD-DXM, confirming its impressive therapeutic effect. Previously, a high initial response was reported in a large study by Cheng et al. [20] after adult ITP patients were treated with a single dose of HD-DXM. The international consensus report and practice guidelines for ITP have now proposed HD-DXM therapy as one of the first-line therapeutic options for ITP patients [23, 24]. The latest clinical investigation showed that in addition to being generally better tolerated, HD-DXM resulted in a higher incidence of overall initial response and complete response compared to prednisone. These findings suggested that HD-DXM may be a preferred corticosteroid strategy for first-line management of adult primary ITP [25].

Although the etiology of ITP is multifactorial, many researchers have shown that ITP involves Th1 polarization, characterized by the oligoclonal accumulation of Th1 cells [3, 26]. Chemokines and chemokine receptors that are involved in T cell differentiation could influence the balance of Th1/Th2 [4]. These results prompted us to investigate the expression of chemokines and their receptors in ITP patients.

CXCL11 is one of the three ligands of CXCR3, the others being CXCL9 and CXCL10. CXCL11 and CXCL10 may play a role in the accumulation of Th1 cells in pulmonary sarcoidosis, a Th1-associated disease [27]. Of the three ligands, CXCL11 has the highest receptor binding affinity for CXCR3 through chemotactic migration and transient mobilization of intercellular calcium [28]. Here, we detected the status of CXCL11 and its receptor CXCR3 in ITP. We found that plasma CXCL11 levels in active ITP patients were higher than in controls, suggesting that CXCL11 may be an important chemoattractant for effector T cells involved in ITP. Moreover, CXCL11 acts as an antagonist for CCR3 but an agonist for CXCR3 [29]. In patients who responded well to pulsed HD-DXM (responders), plasma CXCL11 and mRNA levels of CXCL11 in PBMCs were decreased. Therefore, CXCL11 may be a potential therapeutic target for ITP, since the analogue is that Tizina et al. [30] successively modelled the structural determinants of these interactions of CXCL9/CXCR3, CXCL10/CXCR3 and CXCL11/CXCR3 complexes and their physico-chemical features in order to be used for drug design.

CCL5 is a CC chemokine that activates cells by binding to Th1-associated CCR1 and CCR5 [31]. CCL5/CCR5 interaction has been reported in a number of Th1-associated diseases, such as rheumatoid arthritis, multiple sclerosis [32], human immunodeficiency virus 1 (HIV-1) infection [33], Crohn’s disease [7], and oral lichen planus [8]. In this study, for mRNA levels of CCL5 in PBMCs, no significant difference was found between any group; however, plasma levels of CCL5 in active ITP patients was lower than in controls, perhaps because platelets are the principal source of CCL5 [34]. With the increase of platelet counts after pulsed HD-DXM treatment, there was an increase of CCL5 concentration in plasma. Thus, an important role for CCL5 in the pathogenesis of ITP is not evident; there are possibly other chemokines [35] binding to CCR5 to destroy platelets.

CCL11 is a specific and potent eosinophil chemoattractant that acts exclusively via CCR3 [36]. CCR3 is selectively expressed on Th2 subset, so CCL11 and CCR3 are effectors of the Th2 response. Adzemovic et al. [37] established a congenic rat strain with the *Eae18b* locus containing a chemokine cluster (CCL2, CCL7, CCL11, CCL12, and CCL1) from the experimental autoimmune encephalomyelitis (EAE)- resistant PVG rat strain. The group observed a milder disease and elevated CCL11 mRNA and protein levels in inguinal lymph nodes in the *Eae18b* congenic strain. Increased intrathecal production of CCL11 in congenic rats was accompanied by a tighter blood brain barrier, reflected by more occludin-positive blood vessels. In addition, the congenic strain showed a reduced antigen specific response and a predominant anti-inflammatory Th2 phenotype. Our results suggest that the reduced expression of CCR3 and the decline of CCL11 may be one of the causes of the Th1/Th2 imbalance in ITP. Correspondingly, the restoration of CCR3 and CCL11 after pulsed HD-DXM suggests the rebalancing of Th1/Th2. Thus, upregulation of CCL11 and CCR3 was associated with a Th2-like immune response, which could skew the immune response away from the autoimmune state.

## Conclusions

Our data suggest that polarization of Th1-associated chemokines and chemokine receptors may play important roles in the pathogenesis of ITP. Further, rectifying the abnormal chemokine profile associated with the Th1/Th2 imbalance by treatment with pulsed HD-DXM provides us with new insight into the immunoregulatory mechanisms for the treatment of ITP.

## Abbreviations

ITP: immune thrombocytopenia
Th: T helper
HD-DXM: high-dose dexamethasone
PBMC: peripheral blood mononuclear cell
IL: interleukin
IFN: interferon
EC: ecchymoses
GC: glucocorticoid
PT: petechiae
EP: epistaxis
GUH: genitourinary hemorrhage
GIH: gastrointestinal hemorrhage
GH: gingival hemorrhage
ELISA: enzyme linked immunosorbent assay
PCR: polymerase chain reaction
PBMC: peripheral blood mononuclear cell
CMV: cytomegalovirus
